# *Bacillus subtilis* DNA fluorescent sensors based on hybrid MoS_2_ nanosheets

**DOI:** 10.1371/journal.pone.0297581

**Published:** 2024-02-01

**Authors:** Son Hai Nguyen, Van-Nhat Nguyen, Mai Thi Tran

**Affiliations:** 1 School of Mechanical Engineering, Hanoi University of Science and Technology, Hanoi, Vietnam; 2 College of Engineering and Computer Science, VinUniversity, Hanoi, Vietnam; 3 VinUni-Illinois Smart Health Center, VinUniversity, Hanoi, Vietnam; Federal University of ABC, BRAZIL

## Abstract

Although sensor technology has advanced with better materials, biomarkers, and fabrication and detection methods, creating a rapid, accurate, and affordable bacterial detection platform is still a major challenge. In this study, we present a combination of hybrid-MoS_2_ nanosheets and an amine-customized probe to develop a fast, sensitive biosensor for *Bacillus subtilis* DNA detection. Based on fluorescence measurements, the biosensor exhibits a detection range of 23.6–130 aM, achieves a detection limit of 18.7 aM, and was stable over four weeks. In addition, the high selectivity over *Escherichia coli* and *Vibrio proteolyticus* DNAs of the proposed *Bacillus subtilis* sensors is demonstrated by the fluorescence quenching effect at 558 nm. This research not only presents a powerful tool for *B*. *subtilis* DNA detection but also significantly contributes to the advancement of hybrid 2D nanomaterial-based biosensors, offering substantial promise for diverse applications in biomedical research and environmental monitoring.

## Introduction

*Bacillus subtilis*, a gram-positive bacterium ubiquitously presented in soil and the gastrointestinal tract, plays a pivotal role across various sectors, necessitating its precise detection. As a key player in industrial biotechnology, *B*. *subtilis* is used in producing enzymes, antibiotics, and other biologically active compounds, making its accurate detection crucial for ensuring product quality and safety [1–4]. In the food industry, *B*. *subtilis* detection is vital due to its potential role in food spoilage, especially considering its spore-forming capabilities that allow survival under adverse conditions [5,6]. For the scientific community, *B*. *subtilis* serves as a model organism for genetic and biochemical research, necessitating its precise quantification [7,8]. Moreover, though predominantly non-pathogenic, monitoring *B*. *subtilis* is essential in healthcare settings to circumvent potential infections among immunocompromised individuals [9]. Therefore, the comprehensive role and impact of *B*. *subtilis* necessitate the development of precise and robust detection mechanisms.

The conventional methods to detect *B*. *subtilis* are microbial culture techniques, staining procedures, and molecular methods such as polymerase chain reaction (PCR) [10,11]. Microbial culturing, while a gold standard, often requires a significant duration to yield results, which may not always be compatible with real-time monitoring or immediate needs. Staining procedures, such as the Gram stain, while offering quicker results, may lack specificity. On the molecular front, PCR is a powerful tool for detecting and identifying *B*. *subtilis*. However, it demands sophisticated equipment and technical expertise, challenging routine, and on-field applications. These inherent shortcomings indicate an unmet need for a rapid, specific, and user-friendly method for detecting *B*. *subtilis*, such as a point-of-care device or biosensor.

Biosensors based on nanomaterials have emerged as a compelling alternative in the quest for more efficient, rapid, and reliable methods to detect biological molecules, including DNA, proteins, and cells [12,13]. They have widespread applications in diverse fields, such as clinical diagnosis, environmental monitoring, and food safety. While biosensors can be engineered to detect various signals, their primary function is quantifying a specific entity’s concentration. The scientific community has mainly concentrated on electrochemical and optical biosensors for analyte detection [14,15]. Especially, optical DNA biosensors boast several benefits in biotechnology and medical diagnostics, including high sensitivity and specificity, real-time and label-free detection, and portability [16–18]. As technology progresses, enhancements in these optical biosensors’ sensitivity, specificity, and portability continue to be achieved. Methods to elevate the sensitivity and selectivity of optical DNA sensors include the fabrication of innovative nanomaterials, the development of new sensing platforms, and optimizing sensor preparation parameters.

Recently, the use of molybdenum disulfide (MoS_2_) nanosheets in detecting DNA, including that of *B*. *subtilis*, has gained considerable research attention due to its unique properties, such as electrical, mechanical, and optical characteristics [19–21]. The high surface area of these 2D nanosheets allows for the effective immobilization of probe DNA, facilitating efficient hybridization with target DNA sequences. In addition, hybrid MoS_2_ nanosheets combined with other nanomaterials can further enhance the sensing performance by exploiting the synergistic effects. Hybrid MoS_2_ nanosheets hold several advantages over their pure counterparts, including improved sensitivity, selectivity, stability, and an extended range of analytes that can be detected [22–24]. Thus, applying this hybrid material in optical biosensors represents an intriguing research avenue. In a previous report [25], hybrid MoS_2_ demonstrated a strong capability for *E*. *coli* DNA detection. Here, we develop a new sensing platform using hybrid MoS_2_ nanosheets to detect *B*. *subtilis* DNA within the 23.6–130 aM range. The influence of sensing material concentrations on the sensitivity of a hybrid MoS_2_-based sensor designed for *B*. *subtilis* detection is also investigated. In addition, we examine the selectivity of the proposed sensors over two other bacterial DNA, including *E*. *coli* and *V*. *proteolyticus*, and the stability of the proposed sensors over a month.

## Method and materials

### Chemical and preparation of hybrid MoS_2_ nanosheets

**Fig 1:**
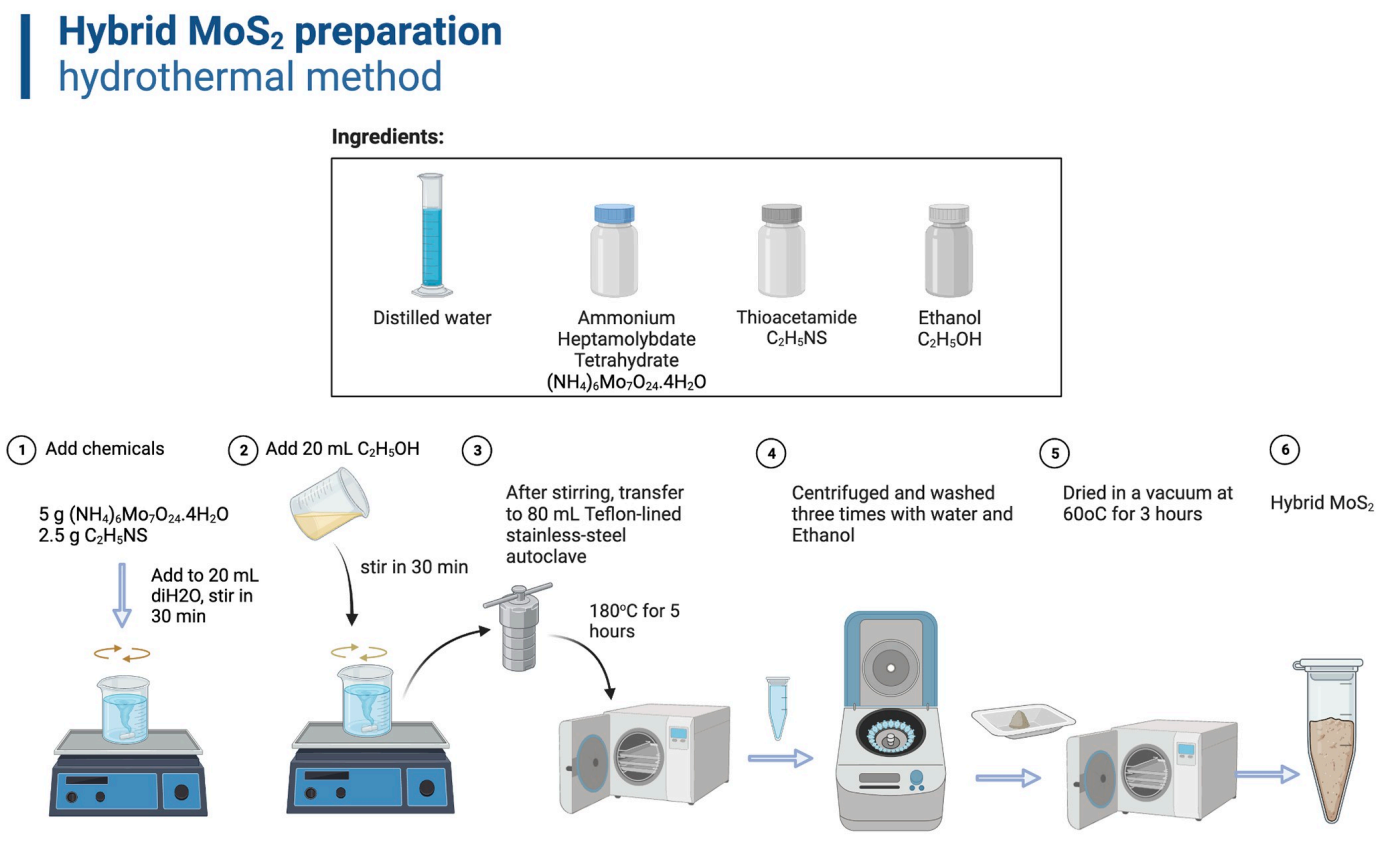
Schematic of hybrid-MoS_2_ nanosheet preparation using hydrothermal method.

The chemicals and preparation methodologies have been thoroughly outlined in the previous study [25] and illustrated in Fig 1. We used the chemicals without any further purification as follows: Ammonium Heptamolybdate Tetrahydrate ((NH_4_)_6_Mo_7_O_24_.4H_2_O, 99.0%, from Tianjin Chemical Reagent Factory, Tianjin, China), Thioacetamide (C_2_H_5_NS, 99.0%, from Shanghai Zhanyun Chemical Co., Ltd, Shanghai, China), Ethanol (C_2_H_5_OH, 99.5%, from Xilong Scientific Co., Ltd., Guangdong, China), and deionized (DI) water. Briefly, we used the hydrothermal method to prepare hybrid MoS_2_ nanosheets. We dissolved and mixed two precursor chemicals of (NH_4_)_6_Mo_7_O_24_.4H_2_O and C_2_H_5_NS in 20 mL of deionized water. After that, 20 mL ethanol was gradually added and stirred for 30 minutes. The solid product was transferred to a 200 mL Teflon-lined stainless-steel autoclave. The hydrothermal temperature was set at 180° C for 5 hours. After this process was done. The precipitation was collected by centrifugation at 5000 rpm, washed with DI water, and dried in a vacuum at 60°C for 3 hours.

### DNA extraction

The *B*. *subtilis* strain was obtained from the Microbiology and Genetics Lab at the Hanoi University of Science and Technology in Hanoi, Vietnam. Starting with 1.5 mL of an overnight *B*. *subtilis* culture grown in Luria Broth (LB) medium, the cells are first pelleted by centrifuging at 8,000×g for 5 minutes using a Hettich Mikro 200R centrifuge (Tuttlingen, Germany). The supernatant is discarded, and the resulting cell pellet is resuspended in 740 μL of TE buffer. Subsequently, 20 μL of 100 mg/mL Lysozyme is added to break down the cell wall, then an incubation occurred at 37°C for 30 minutes. Next, 40 μL of 10% SDS and 8 μL of Proteinase K (10 mg/mL) (all from Biobasic, Canada) are introduced, assisting in protein digestion and membrane disruption. After a further incubation at 56°C for 3 hours, 100 μL of 5 M NaCl and heated CTAB/NaCl (from Merck, Germany) at 65°C are added sequentially to promote DNA precipitation. After an incubation at 65°C for 10 minutes, the sample undergoes a chloroform: isoamyl alcohol extraction (from Sigma Aldrich) to separate the DNA from impurities. After centrifuging at 12,000×g for 10 minutes at room temperature, the aqueous phase containing the DNA is transferred to a new tube. This extraction step is repeated until no white protein layer is visible. The DNA is then precipitated using cold 100% ethanol (Merck, Germany) and incubated at -20°C for 2 hours overnight. After additional centrifugation at 12,000×g for 15 minutes at 4°C, the DNA pellet is washed with 50 μL of 70% ethanol to remove salts and other impurities. Once the pellet dries, it’s resuspended in the TE buffer for storage. Ideally, the isolated DNA should be stored at −20°C for future use. All the DNA utilized in this study was evaluated using the OD260/280 ratios using a DeNovix UV-Visible spectrometer (Model: DS-11 FX+), yielding results around 2.0, indicating the high purity of the DNA samples.

### Measuring the optical properties of *B*. *subtilis* DNA sensors based on hybrid MoS_2_ nanosheets

This study utilized an oligonucleotide probe with the sequence amine—5’-CCTACGGGAGGCAGCAGTAG-3’, complementary to *B*. *subtilis* DNA [8]. The probe was diluted to 30 nM in TE buffer in all measurements. DNA solutions were prepared by dissolving and diluting them in 1×TE buffer (10 mM Tris-HCl, 1 mM EDTA, pH 8.0). *B*. *subtilis* DNA was pretreated using the heating method, which involved heating the samples at 95°C for 30 minutes. For a specific test, designated concentrations of the probe and hybrid MoS_2_ nanosheets were utilized to determine the sensors’ absorbance and photoluminescence (PL). We incorporated 900 μL of hybrid MoS_2_ into a 10 mm cuvette, with TE buffer as the solvent. We then added 100 μL of the probe and gradually added 100 μL DNA to the cuvette to create concentrations ranging from 23.6 to 130 aM. At each step, PL measurements were performed. The fluorescence intensities at an excitation wavelength of white light, using a slit width of 300 μm and an exposure duration of 1 second, were recorded. To investigate the effects of the sensing materials, the experiment is repeated with varying concentrations of hybrid MoS_2_ (10 mg/L, 20 mg/L, 30 mg/L, 40 mg/L, and 50 mg/L).

## Results and discussions

### Characterizations of prepared materials

**Fig 2:**
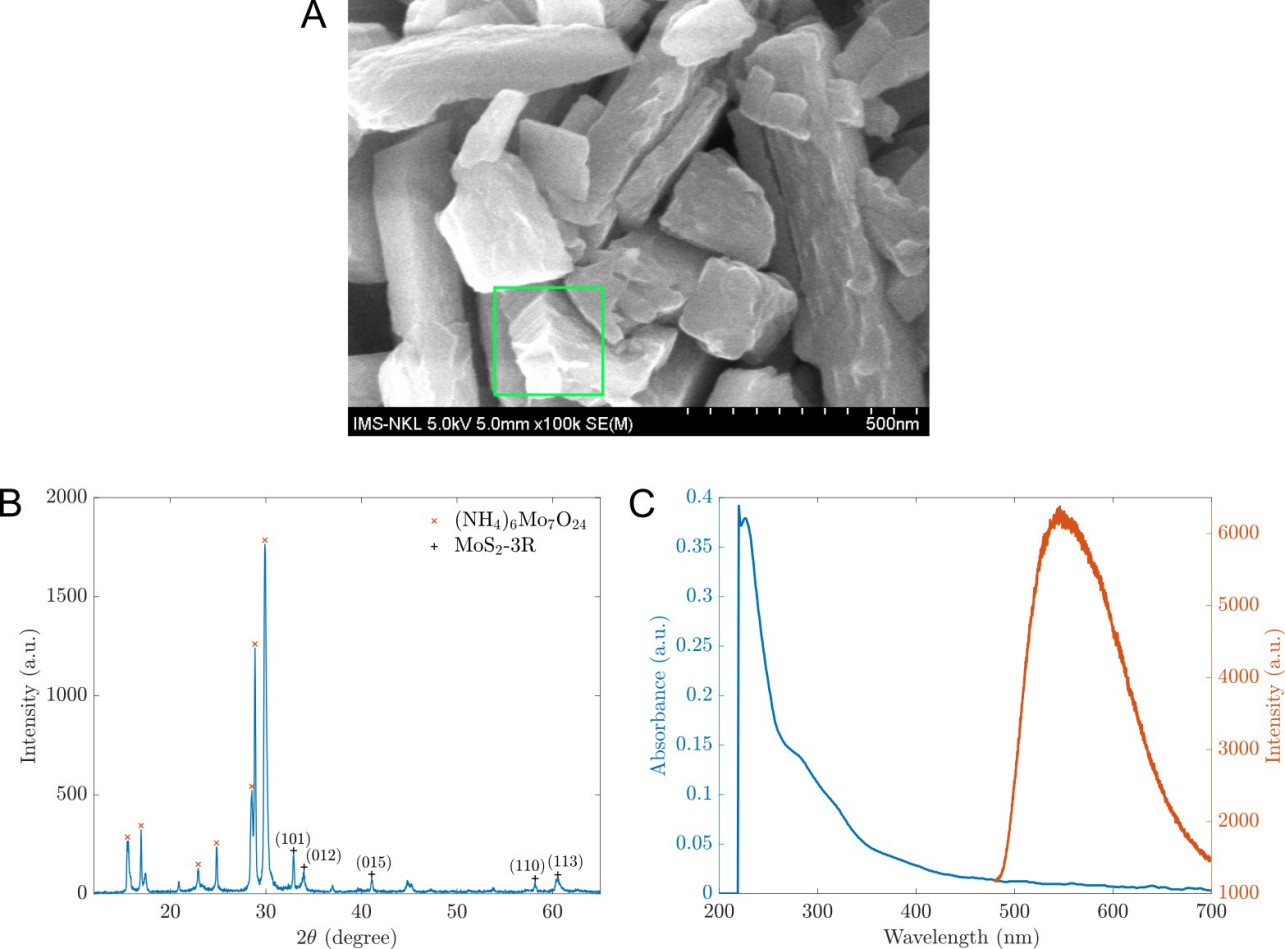
The characteristics of the prepared materials. (A) The SEM image captured by HITACHI-S4800 confirms the nanosheet morphology (inside the green region of interest); the contrast was enhanced. (B) The XRD pattern obtained by Rigaku MiniFlex600, and (C) The absorbance and photoluminescence attributes. Absorbance (blue line) was determined using a DeNovix UV-Visible spectrometer (Model: DS-11 FX+). The fluorescence intensities (orange line) were recorded using a spectrophotometer with a 10 nm slit-width (SpectraPro HRS-300, Teledyne Princeton Instruments, Trenton, NJ 08619 USA) at an excitation wavelength of white light and an exposure duration of 1 second.

The produced materials were initially assessed for their structure and morphology using XRD and SEM imaging techniques. As illustrated in Fig 2A, these materials reveal a nanosheet structure. The XRD pattern, displayed in Fig 2B, presents five unique peaks at positions (101), (012), (015), (110), and (113), suggestive of the MoS_2_-3R structure (PDF#17–0744, as analyzed with JADE software by MDI Materials Data). The resulting composite was also detected alongside MoS_2_-3R, (NH_4_)_6_Mo_7_O_24_. However, the SEM image, shown in Fig 2A, emphasizes the existence of multilayer nanosheets within the hybrid material. This observation suggests that (NH_4_)_6_Mo_7_O_24_ plays a critical role in creating the lamellar MoS_2_, with NH_4_^+^ ions occupying the layers in between and indicates that (NH_4_)_6_Mo_7_O_24_ either functionalizes the MoS_2_ surface or decomposes into molecules. Furthermore, we explored the optical properties of the manufactured materials. An absorbance peak at 235 nm and a photoluminescence peak at 558 nm can be observed in Fig 2C.

### Direct detection of *B*. *subtilis* DNA using fluorescent sensors based on the hybrid MoS_2_ nanosheets

**Fig 3:**
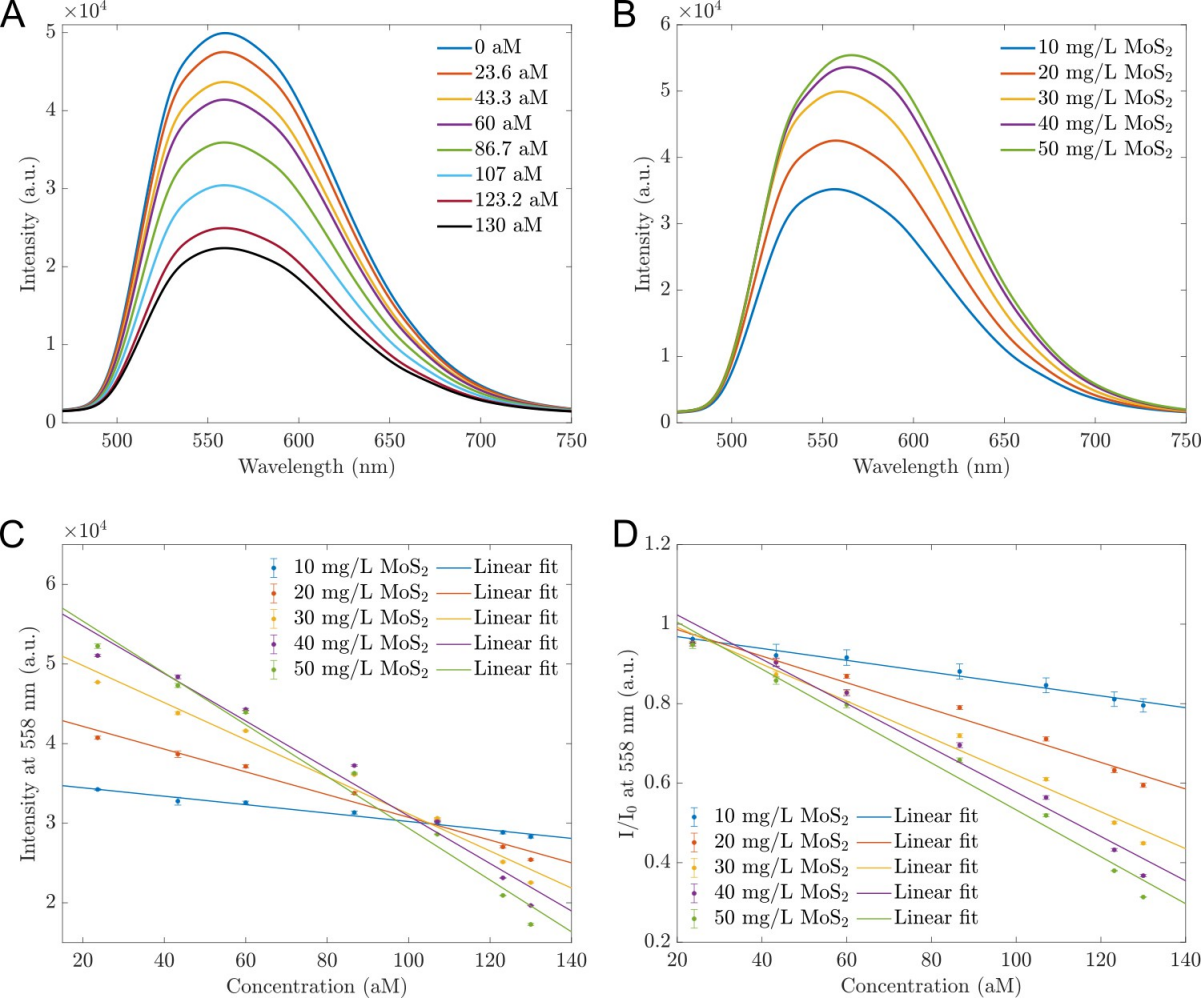
PL spectra of sensors exposed to different concentrations of *B*. *subtilis* DNA. (A) PL spectra of sensors based on 30 mg/L MoS_2_ while in contact with various *B*. *subtilis* DNA concentrations. When the DNA concentrations increased, the maximum intensities decreased. (B) The PL intensities of different sensors with multiple concentrations of hybrid MoS_2_ nanosheets before in contact with *B*. *subtilis* DNA; The higher the sensing material concentration, the higher the PL. (C) The dependence of the intensities *I* at 558 nm of different sensors on the *B*. *subtilis* DNA concentrations. This relationship can be described by a linear function. (D) The ratios *I*/*I*_0_ derived from the intensity at 558 nm of different sensors depending linearly on the DNA concentrations, where *I*_0_ is the intensity of the sensor before contact with DNA. The error bars represent for standard deviations of nine measurements.

This study aims to design a simple optical sensing platform to detect a range of *B*. *subtilis* DNA concentrated from 23.6 to 130 aM, reflected by the number of copies of the testing sample from 16×10^6^ to 16×10^7^. In our experiment, the probe concentration was 30 nM (using 100 μL, which contains 1.83×10^12^ copies, a much more considerable amount than the number of ssDNA copies) and the concentration of sensing materials was varied. The sensors’ fluorescence was examined at hybrid MoS_2_ concentrations of 10 mg/L, 20 mg/L, 30 mg/L, 40 mg/L, and 50 mg/L, and was presented in Fig 3.

Fig 3A shows an example of PL spectra of sensors based on 30 mg/L hybrid MoS_2_ nanosheets in contact with *B*. *subtilis* DNA. The other MoS_2_ concentration sensors had similar shapes and quenching effects with DNA concentrations increased. They all had fluorescent peaks at 558 nm. The fluorescence spectra of all sensors based on 10 mg/L to 50 mg/L hybrid MoS_2_ before adding DNA are presented in Fig 3B. Based on the intensity *I* and the ratio *I*/*I*_0_ at the wavelength 558 nm, we established the calibration lines of *I* vs *C* and *I*/*I*_0_ vs. *C* in Fig 3C & 3D, where *I* is the intensity of sensors with specific concentration of DNA; *I*_0_ is the initial fluorescent intensity of sensors; *C* is the concentration of *B*. *subtilis* (aM). The operating functions were estimated and shown in Table 1. All sensors can operate linearly with high precision (all values of R^2^ were about 0.98). Among them, the sensors based on 50 mg/L have the highest sensitivity with the highest slope of -325. However, the 30 mg/L MoS_2_-based sensors have the best detection limit (LOD) of 18.7 aM and high sensitivity (slope of -233.8). In Table 1, the equation determined the limit of detection is LOD = blank signal + 3 standard derivations. Overall, the detection limits are slightly different, about 19 aM. That might be because the sensing material’s concentration is not much different (10 mg/L to 50 mg/L, only 40 mg/L difference).

**Table 1:** Linear relationship between intensity values at 558 nm and *B*. *subtilis* DNA concentrations. *B*. *subtilis* DNA concentrations changed from 23.6 aM– 130 aM of five sensors with concentrations of hybrid MoS_2_ nanosheets of 10 mg/L, 20 mg/L, 30 mg/L, 40 mg/L and 50 mg/L. *C* is the concentration of *B*. *subtilis* DNA (aM).

Hybrid MoS_2_ nanosheets concentration (mg/L)	Intensity at 558 nm (*I*)	*I*/*I*_0_ at 558 nm (*I*/*I*_0_)	R^2^	Concentration LOD (aM)
10	-53.0*C* + 35500	-0.00149*C* + 0.998	0.978	19.8
20	-143*C* + 45000	-0.00334*C* + 1.05	0.98	19.2
30	-233.8*C* + 54500	-0.00464*C* + 1.08	0.981	18.7
40	-298*C* + 60700	-0.00557*C* + 1.13	0.975	21.3
50	-325*C* + 61900	-0.0059*C* + 1.12	0.98	19.1

**Fig 4:**
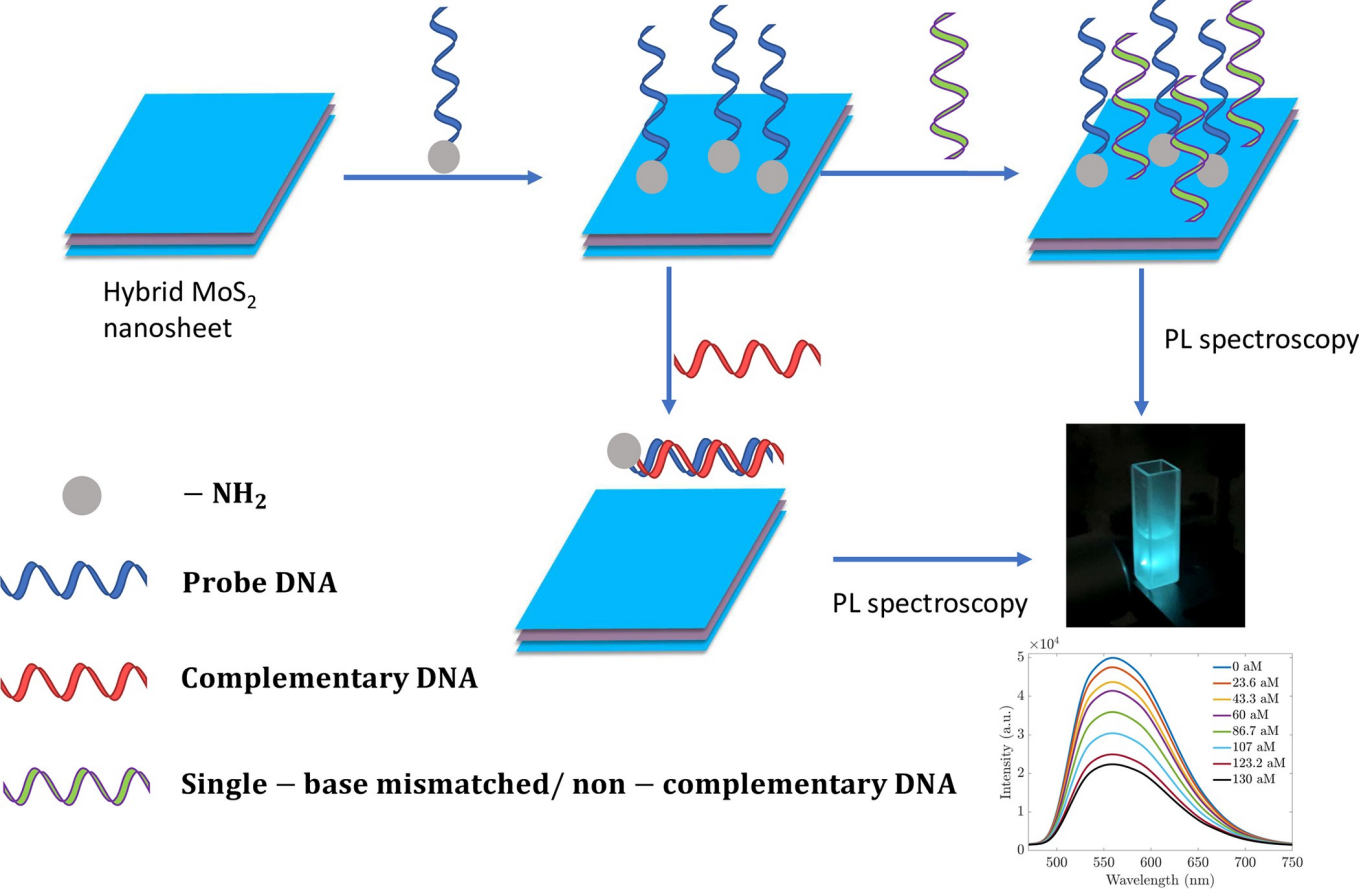
Schematic of experimental procedure and working mechanism of photoluminescence measurements of *B*. *subtilis* DNA sensors.

In all experiments, the quenching effects have occurred and are reflected in the negative slopes of all operating functions. The quenching effect can be explained by the fact that in this study, we employed the NH_2_-5′-CCTACGGGAGGCAGCAGTAG-3′ probe to identify the complementary target *B*. *subtilis* DNA, adhering to the Watson-Crick base-pairing principles [8]. The probe was chemically modified with an amine group (NH_2_) to enhance bonding to the MoS_2_ surface. In the prepared nanomaterials, NH_4_^+^ ions occupying the layers in between either functionalize the MoS_2_ surface or help to bind with NH_2_- of the amine probe. Fig 4 provides a proposed schematic of the adsorbed single-stranded DNA (ssDNA) on the hybrid MoS_2_ surface. The ssDNA can adhere to this surface, altering the dielectric properties of MoS_2_. However, when the ssDNA hybridizes with its complementary DNA, the resultant double-stranded DNA (dsDNA) establishes poor contact with the hybrid MoS_2_, distancing itself from the MoS_2_ surface and changing the dielectric environment from DNA to water and reducing the photoluminescence [25]. The more complementary ssDNA added to the sensor, the more dsDNA was formed. Consequently, the photoluminescence was reduced more intensively. The intensity *I* at 558 nm, and the ratio of *I*/*I*_0_ at 558 nm can be used to estimate the unknown *B*. *subtilis* DNA concentrations.

**Fig 5:**
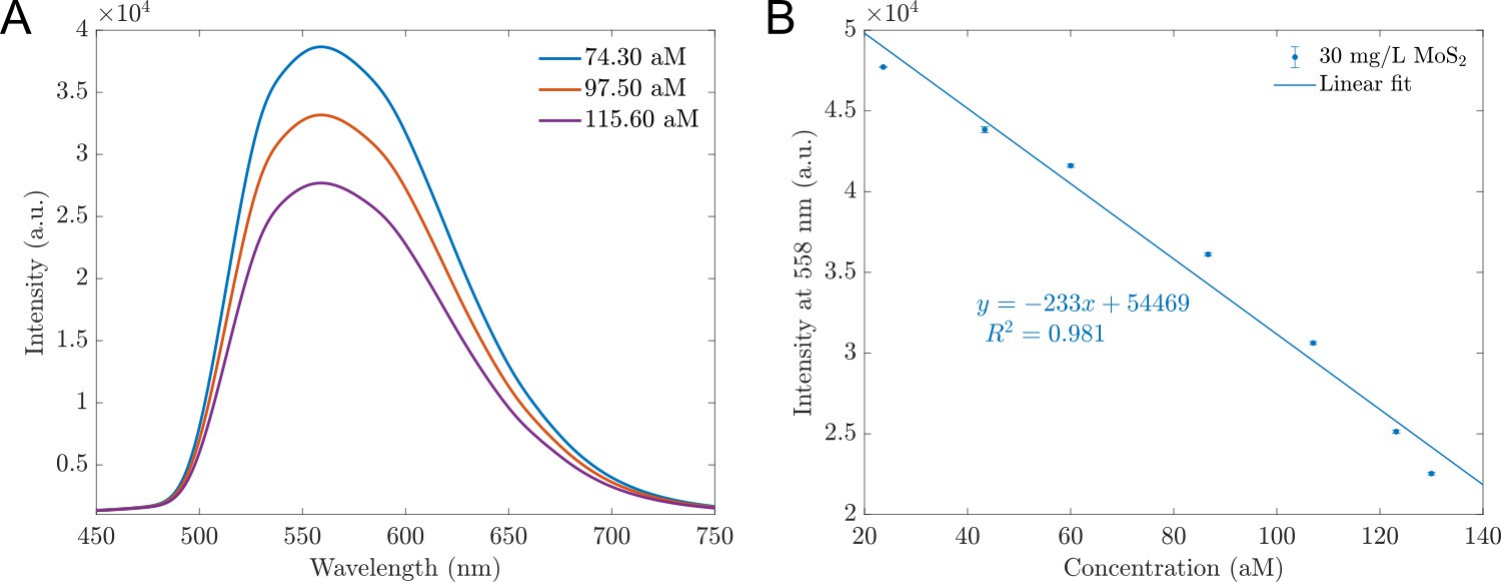
Validation of the operational performance of the proposed sensor. (A) The fluorescence spectra of testing DNA samples using the proposed sensors, including 74.3 aM, 97.5 aM, and 115.6 aM. (B) The fitting line of proposed sensors derived from the fluorescence of 30 mg/L MoS_2_-based sensors at 558 nm from Fig 3A. This operating line will refer to the calibrated concentrations for test samples. The error bars represent the standard deviations of nine measurements.

To validate the operational performance of this sensor, we prepared various DNA concentrations exceeding the limit of detection (LOD) and within the range defined by the calibration line. The concentrations examined were 74.3 aM, 97.5 aM, and 115.6 aM. The corresponding fluorescence measurements are depicted in Fig 5A. Utilizing the fluorescence values at 558 nm into the calibration line in Fig 5B, we determined the measured concentrations listed in Table 2. The calibrated concentrations were in good agreement with the experimental concentrations with small percentages of difference (smaller than 10%). When the test sample had a high concentration, the measured intensity was higher, and the precision improved. For example, with the sample of 115.6 aM, the difference between the actual concentration and the calibrated one was only 1.52%. The result confirms the reliability and repeatability of our proposed sensor.

**Table 2:** Validation of the designed sensing platform derived from Fig 5.

Testing concentration (aM)	Fluorescence at 558 nm	Estimated concentration (aM)	Error (%)
74.30	38864.89 ± 95.00	66.87	9.99
97.50	33371.11 ± 88.90	90.37	7.31
115.60	27882.22 ± 98.49	113.85	1.52

### Selectivity and stability of the proposed sensors

**Fig 6:**
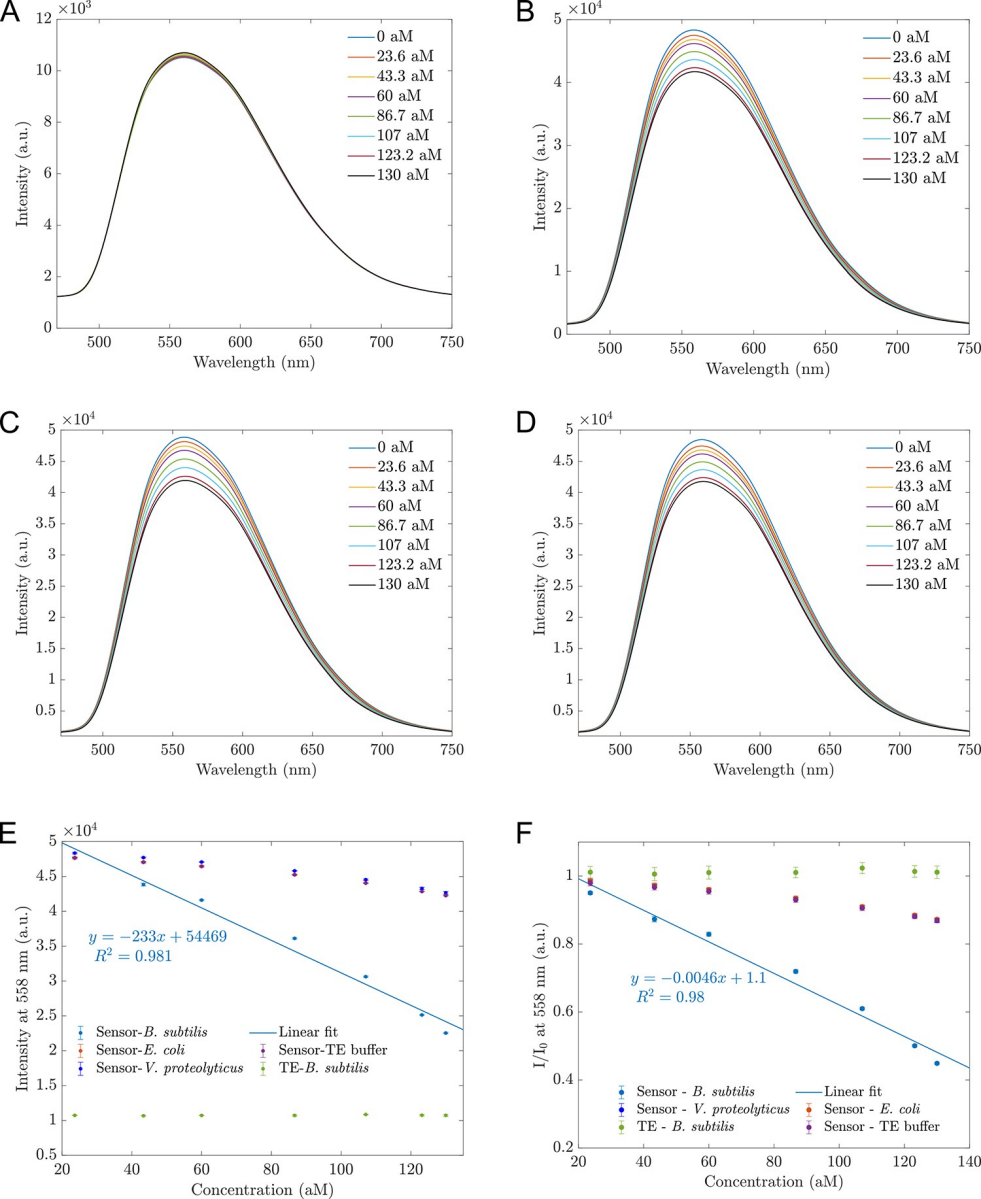
The selectivity of the proposed sensors. (A) Fluorescence spectra of different *B*. *subtilis* DNA concentrations; (B) Fluorescence spectra of 30 mg/L hybrid MoS_2_ based on the sensors when adding TE buffer. Legends represent added volumes equivalent to DNA concentrations; (C) Fluorescence spectra of proposed sensors in contact with *V*. *proteolyticus* DNA; (D) Fluorescence spectra of proposed sensors in contact with *E*. *coli* DNA; (E) Intensity changes at 558 nm corresponding to varying concentrations of added analytes derived from Fig 3A (for *B*. *subtilis* DNA) and Fig 6A–6D; (F) Ratios of *I*/*I*_0_ at 558 nm change with the concentrations of added analytes derived from Fig 3A (for *B*. *subtilis* DNA) and Fig 6A–6D. *I*_0_ is the fluorescence of sensors before adding DNA or TE. Error bars represent standard deviations calculated from 9 measurements.

In this section, the selectivity and stability of the proposed sensors are investigated. First, we recorded the fluorescence of *B*. *subtilis* DNA of concentration from 23.6 to 130 aM. The fluorescences when the proposed sensors were in contact with various TE buffer concentrations, were also measured. Fig 6A showed that the tested *B*. *subtilis* DNA concentrations had low fluorescence and were almost the same intensity of 1.1×10^4^. Hence, our proposed sensors’ fluorescence changes in contact with *B*. *subtilis* DNA were not due to the changes in *B*. *subtilis* DNA fluorescence. To confirm the feasibility and selectivity of the proposed sensors, we prepared sensors based on 30 mg/L hybrid MoS_2_ nanosheets. These sensors were exposed to different analytes, including TE buffer, *Vibrio proteolyticus* DNA, and *Escherichia coli* DNA. The fluorescence spectra in Fig 6B–6D reveal that the spectra and the changes in intensity for TE buffer and mismatched DNAs (*V*. *proteolyticus* and *E*. *coli* DNA) were nearly identical and slightly different. The intensity was reduced from 4.7×10^4^ to 4.0×10^4^ for three analytes. In contrast, the intensity decreased significantly when the target was *B*. *subtilis* DNA from 4.7×10^4^ to 2.0×10^4^ (see Fig 3A). This suggests that the *E*. *coli* and *V*. *proteolyticus* DNA did not induce any changes and merely diluted the hybrid MoS_2_ suspension, mirroring the effect of added TE buffer. This observation aligns with Fig 6E & 6F, where the intensity *I* or ratio *I*/*I*_0_ at 558 nm for TE, *V*. *proteolyticus*, and *E*. *coli* overlapped and changed slightly. *B*. *subtilis* DNA concentration can be determined by the linear function, as shown in Fig 6, within a range of 23.6–130 aM with high precision (R^2^ = 0.98).

For better visualization, the quenching effects were quantified by:

$$Quenching\;(\%)=\frac{I_{0}-I}{I_{0}}\times 100\%$$ (1)

**Fig 7:**
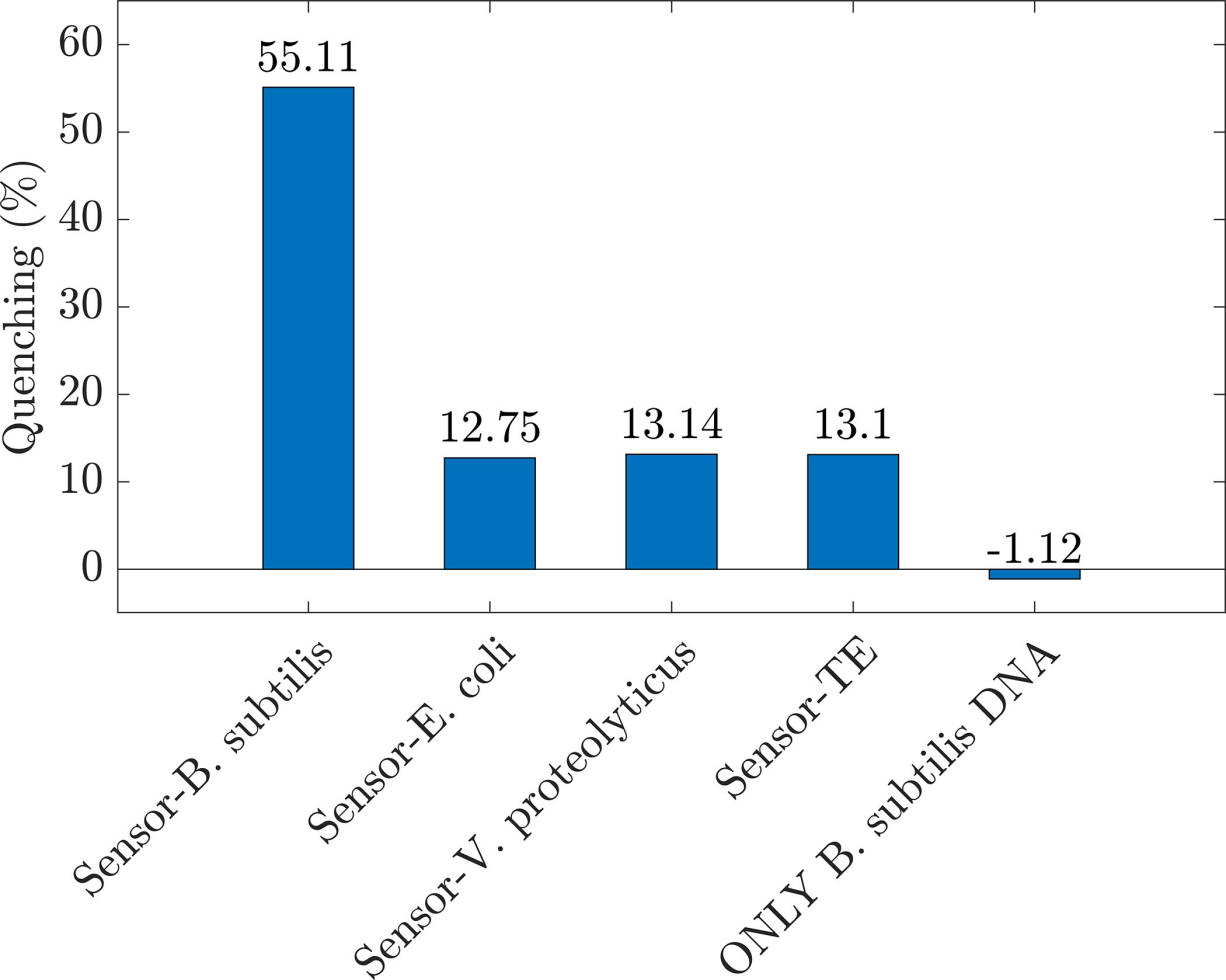
The quenching effect of the sensors. The bar plots illustrated the intensity changes at 558 nm between the analyte concentration of 0 aM and 130 aM. Four bars labeled with sensor—*B*. *subtilis*, sensor—*E*. *coli*, sensor—*V*. *proteolyticus*, and sensor—TE represent the proposed sensors’ experiments in contact with *B*. *subtilis* DNA, *E*. *coli* DNA, *V*. *proteolyticus* DNA, and TE buffer, respectively. The bar labeled with ONLY *B*. *subtilis* DNA was represented for fluorescence of DNA itself without the presence of the proposed sensors.

Fig 7 illustrates the quenching effect of the sensors when 130 aM analytes compared to the sensors’ photoluminescence before adding analytes. In the case of "ONLY *B*. *subtilis* DNA" the reference photoluminescence was the TE buffer. The quenching percentage of *B*. *subtilis* DNA was -1.12%, which means when adding DNA, the fluorescence enhanced. This enhancement is reasonable because the more DNA added, the higher the fluorescence was. However, with the maximum concentration of DNA, the increased intensity was deficient. The non-complementary DNA and TE buffer induced almost the same quenching percentage of 13%, representing the same diluting effect of adding these analytes. Only *B*. *subtilis* DNA, as we discussed above, had a significant quenching (%) of 55%. These findings validate that the proposed fluorescence sensors are functional and offer high sensitivity, reliability, and selectivity.

**Fig 8:**
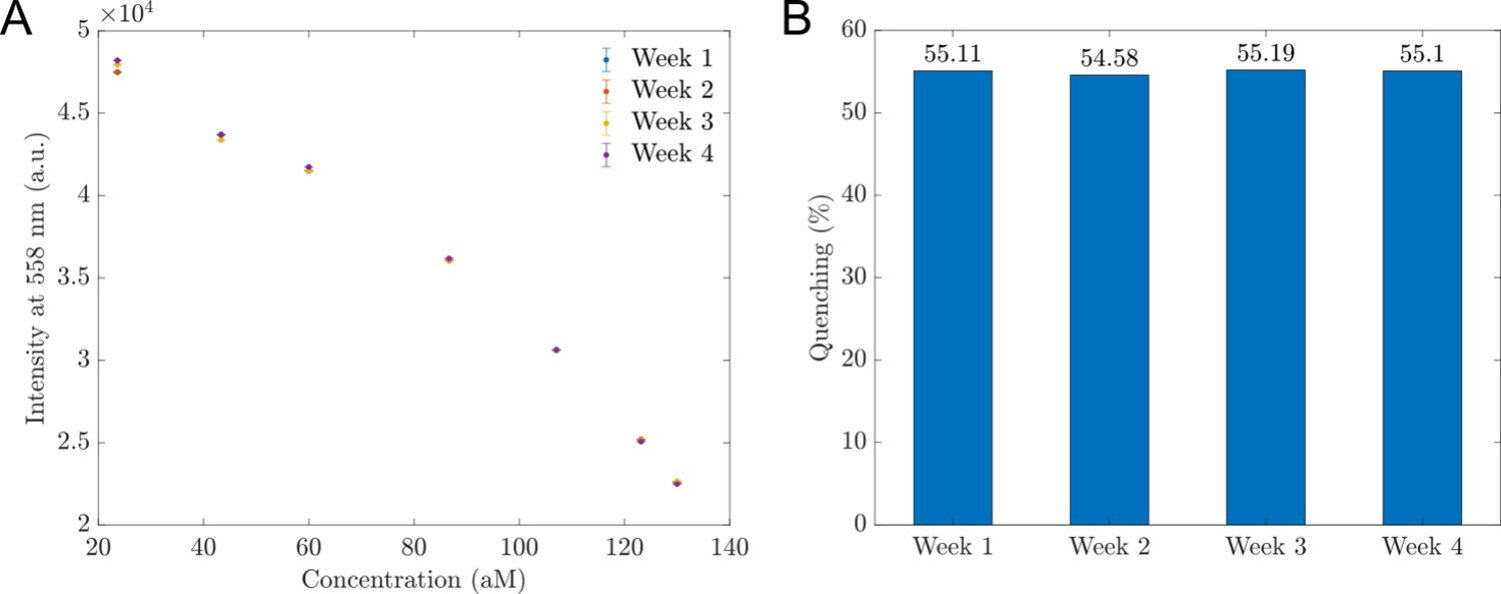
The stability of the proposed sensors. (A) The intensity of the proposed sensors at the wavelength of 558 nm changed to the concentrations of *B*. *subtilis* DNA over a month. (B) The quenching (%) histogram between the PL intensity at 558 nm wavelength of 130 aM sample and 0 aM sample over four weeks.

Furthermore, we explored the stability of the proposed sensors by repeating the tests of PL measurements of 30 mg/L hybrid MoS_2_-based sensors in contact with *B*. *subtilis* DNA every week over 4 weeks. The results of intensities at 558 nm depending on the DNA concentrations and quenching percentage were shown in Fig 8A and 8B, respectively. As observed from Fig 8A and 8B, the intensity of proposed sensors at 558 nm wavelength and quenching percentage were stable over four weeks. These results confirmed the high stability of our proposed sensors to *B*. *subtilis* DNA over time.

Our findings revealed that the relatively straightforward arrangement of an amine probe-hybrid MoS_2_ nanosheets demonstrated significant promise for the detection of *B*. *subtilis* DNA. The proposed sensors offered impressive sensitivity, stability, and specificity, with a detection range of 23.6 to 130 aM and a detection limit of 18.7 aM for 30 mg/L hybrid MoS_2_ nanosheet-based sensors. Notably, there’s been a lack of literature about *Bacillus* DNA sensors. The existing biosensors either have a higher detection limit, are built on costlier materials, and involve more intricate procedures. For instance, Fei Chen et al. designed optical biosensors to identify *B*. *subtilis* DNA with a detection limit of 10^5^ CFU/mL, utilizing a combination of alkaline phosphatase/graphene oxide nanoconjugates and D-glucose-6-phosphate-functionalized gold nanoparticles [26]. Ivan Magnrina’s team presented a novel dual electrochemical genosensor for simultaneously amplifying and detecting *Bacillus anthracis* DNA, with a detection limit of 0.8 fM [27]. Zahra Izadi formulated an electrochemical DNA-based biosensor for *Bacillus cereus* detection employing an Au-nanoparticle-modified pencil graphite electrode with a detection limit of 9.4×10^−12^ M [28]. Mukhil Raveendran produced an electrochemical DNA biosensor to identify *Bacillus anthracis*, which leveraged a thiol probe anchored on gold-modified screen-printed electrodes and had a detection limit of 10 pM [29]. Furthermore, the utilization of hybrid MoS_2_ nanosheets is still in its infancy and remains largely untapped. Based on the authors’ understanding, there’s no documented evidence of using the innovative hybrid-MoS_2_ nanosheets and (NH_4_)_6_Mo_7_O_24_ materials to detect *B*. *subtilis* DNA. Our findings will likely pave the way for further research in pathogen detection applications, which are still in the nascent stages of development.

## Conclusion

In conclusion, this study highlights the successful synthesis of novel hybrid-MoS_2_ nanosheets and their implementation in a sensor platform for detecting *B*. *subtilis* DNA. The sensor platform, comprising hybrid-MoS_2_ nanosheet-amine customized probe-*B*. *subtilis* DNA, demonstrated the ability to detect *B*. *subtilis* DNA within a range of 23.6–130 aM and a detection limit of 18.7 aM. The performance of the sensor platform was evaluated by altering the sensing material concentrations. Optimal conditions for the proposed sensors were determined as MoS_2_ at a concentration of 30 mg/L. The findings reveal that this sensing platform holds significant potential for fluorescence-based sensors, exhibiting high sensitivity, stability, specificity, and precision. This research advances hybrid 2D nanomaterial-based biosensors with potential biomedical research and environmental monitoring applications.
